# Supplementary material for: Cryo-EM reveals evolutionarily conserved and distinct structural features of plant CG maintenance methyltransferase MET1
Source: Nat Commun. 2025 Sep 26;16:8524. doi: 10.1038/s41467-025-63765-9 (PMC12474915; doi:10.1038/s41467-025-63765-9)
Supplement: Supplementary file 1 — Supplementary Information [file 41467_2025_63765_MOESM1_ESM.pdf]

Supplementary Information for

Cryo-EM Reveals Evolutionarily Conserved and Distinct Structural Features of Plant CG Maintenance Methyltransferase MET1

Amika Kikuchi, Atsuya Nishiyama, Yoshie Chiba, Makoto Nakanishi, Taiko Kim To, Kyohei Arita

# Correspondence: aritak@yokohama-cu.ac.jp

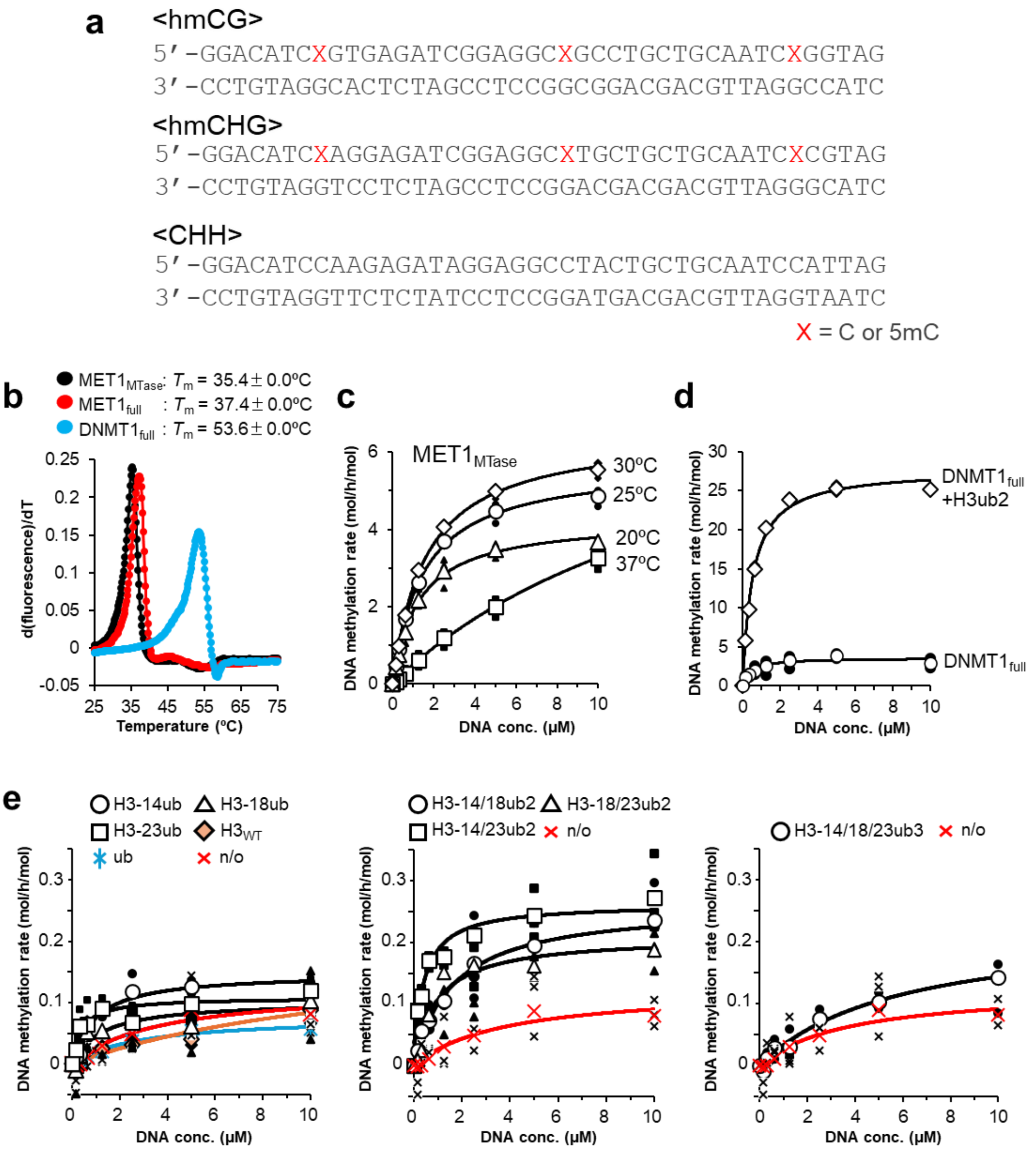

Supplementary Figure 1: Biochemical assays.

(a) DNA sequence using *in vitro* DNA methylation assay as a substrate. **(b)** Thermal stability assays for MET1$_{full}$ (red), MET1$_{MTase}$ (black), and DNMT1$_{full}$ (cyan) proteins. Melting curves were plotted from 25°C to 75°C. Denaturation temperatures of MET1$_{full}$, MET1$_{MTase}$, and DNMT1$_{full}$ are 37.4 ± 0.0ºC, 35.4 ± 0.0ºC, and 53.6 ± 0.0ºC, respectively. Three independent experiments were conducted for each sample. **(c)** *In vitro* DNA methylation assay using MET1$_{MTase}$ at the indicated temperatures. **(d)** *In vitro* DNA methylation activity of DNMT1 in the presence and absence of the H3K18ub/K23ub analog. **(e)** *In vitro* DNA methylation activity of MET1$_{full}$ in the presence of the indicated ubiquitin, histone H3 tail, and ubiquitinated H3 analogs. Data in (b–e) are presented as mean ± SD from three independent biological replicates.

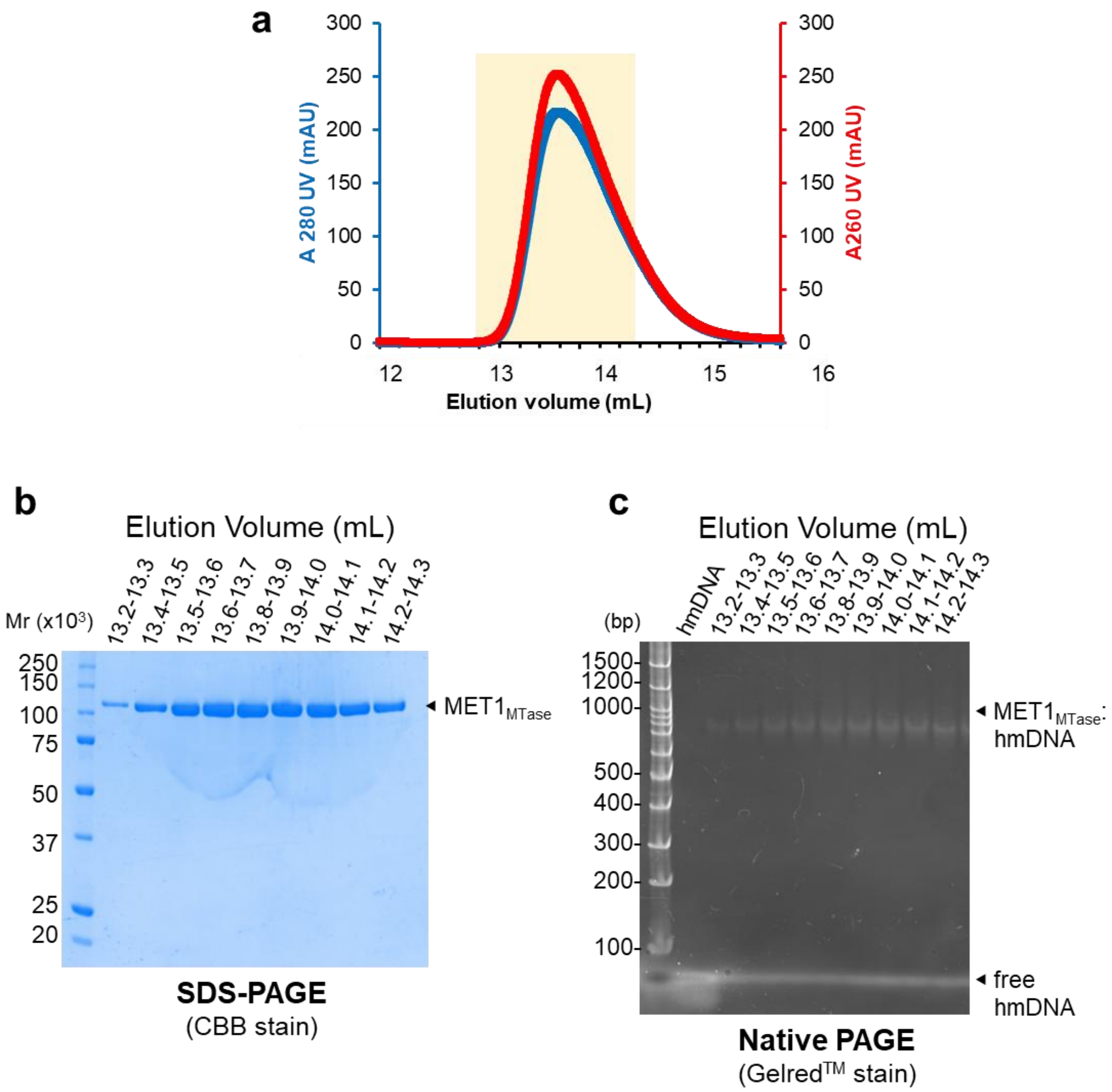

Supplementary Figure 2: Sample preparation of MET1$_{MTase}$ bound to DNA containing mCG/fCG site.

(a) Size exclusion chromatography of a mixture of MET1$_{MTase}$ and hmDNA. All fractions in the chromatography peaks were analyzed by **(b)** SDS-PAGE stained by CBB for detecting proteins and **(c)** Native-PAGE stained by Gelred^{TM} for detecting DNA.

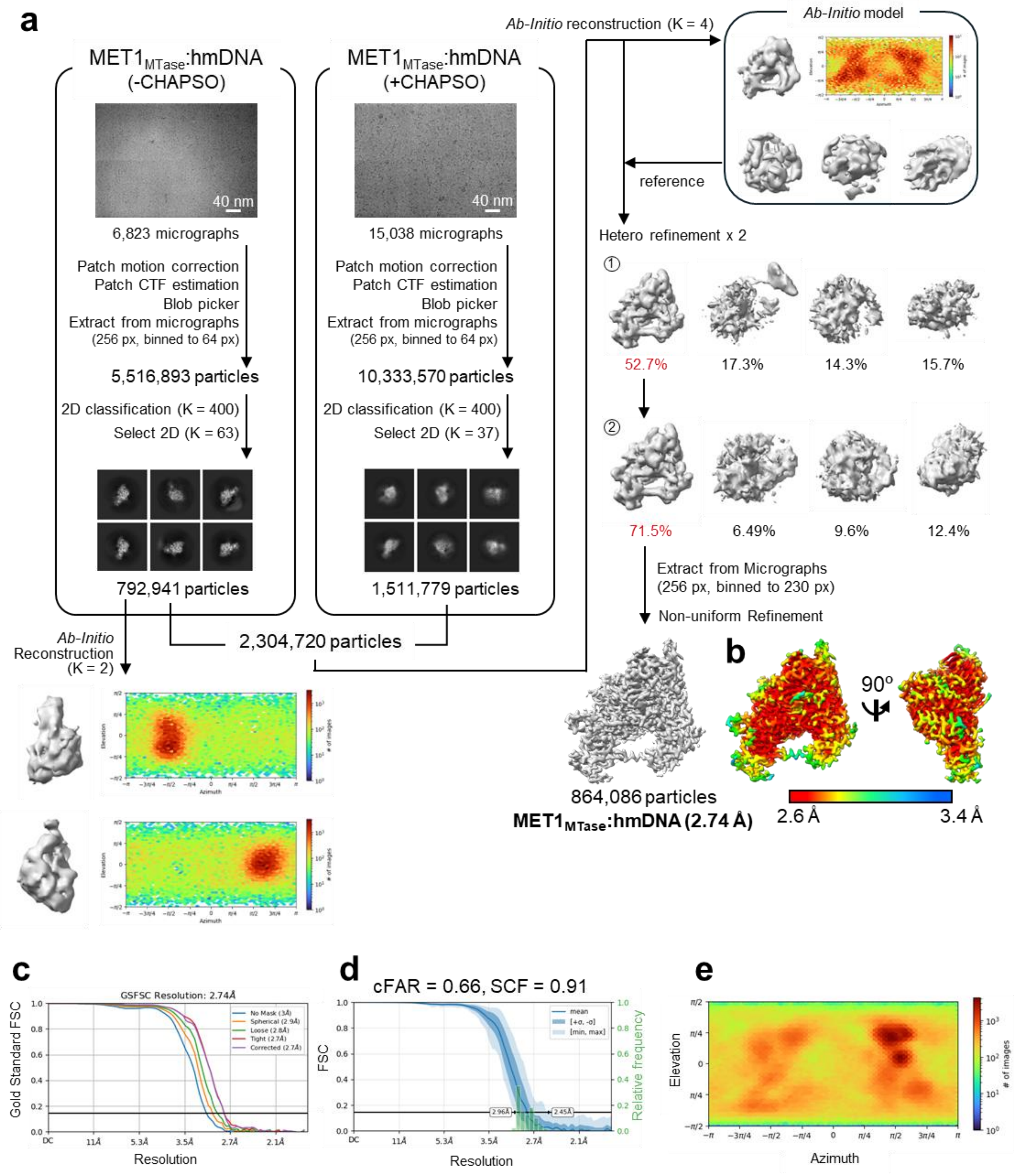

a
MET1MTase:hmDNA (-CHAPSO)
MET1MTase:hmDNA (+CHAPSO)
40 nm
6,823 micrographs
15,038 micrographs
Patch motion correction
Patch CTF estimation
Blob picker
Extract from micrographs
(256 px, binned to 64 px)
5,516,893 particles
10,333,570 particles
2D classification (K = 400)
Select 2D (K = 63)
Select 2D (K = 37)
792,941 particles
1,511,779 particles
2,304,720 particles
Ab-Initio Reconstruction (K = 2)
Ab-Initio reconstruction (K = 4)
Ab-Initio model
reference
Hetero refinement x 2
52.7% 17.3% 14.3% 15.7%
71.5% 6.49% 9.6% 12.4%
Extract from Micrographs
(256 px, binned to 230 px)
Non-uniform Refinement
864,086 particles
MET1MTase:hmDNA (2.74 Å)
b
90°
2.6 Å
3.4 Å
c
GSFSC Resolution: 2.74Å
Gold Standard FSC
Resolution
No Mask (3Å)
Spherical (2.9Å)
Loose (2.8Å)
Tight (2.7Å)
Corrected (2.7Å)
d
cFAR = 0.66, SCF = 0.91
FSC
Relative frequency
mean
2.96Å
2.45Å
Resolution
e
Elevation
Azimuth
# of images

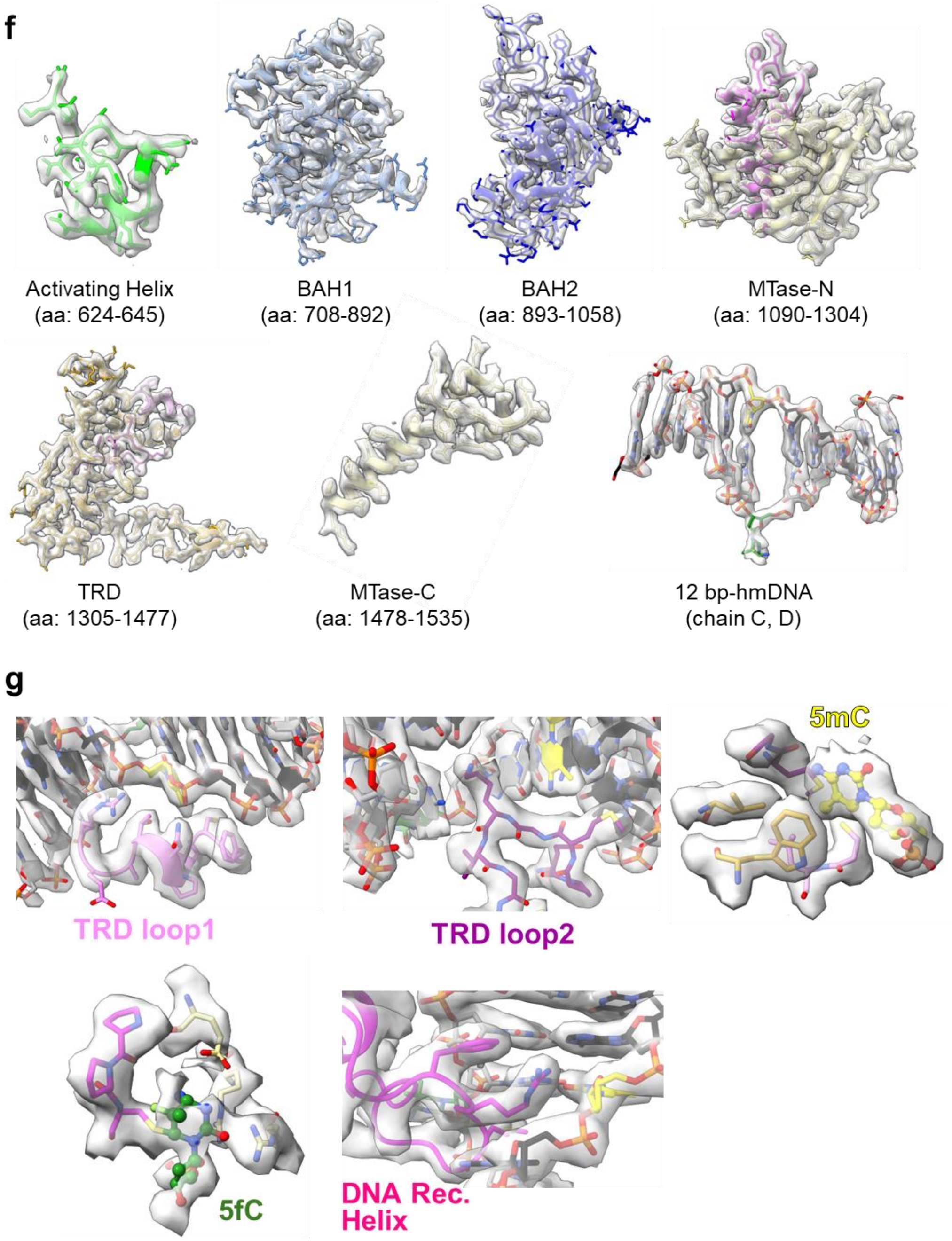

Supplementary Figure 3: Overview of cryo-EM data processing workflow for $MET1_{MTase}$ bound to DNA containing mCG/fCG site.

(a) Data processing workflow performed using cryoSPARC. **(b)** Local resolution map. Resolution ranges are shown in box. **(c)** Fourier Shell Correlation (FSC) curve. Resolution is reported at the FSC threshold of 0.143. **(d)** Directional resolution and Fourier sampling of the cryo-EM map. The conical FSC, the conical FSC area ratio (cFAR), and the sampling compensation factor (SCF) were calculated and plotted using cryoSPARC. **(e)** The orientation distribution of the refined particles. **(f)** Atomic models of MET1 domains and DNA were superimposed on the cryo-EM map (grey). **(g)** A close-up view of the interaction between MET1 and DNA superimposed on the semi-transparent cryo-EM map.

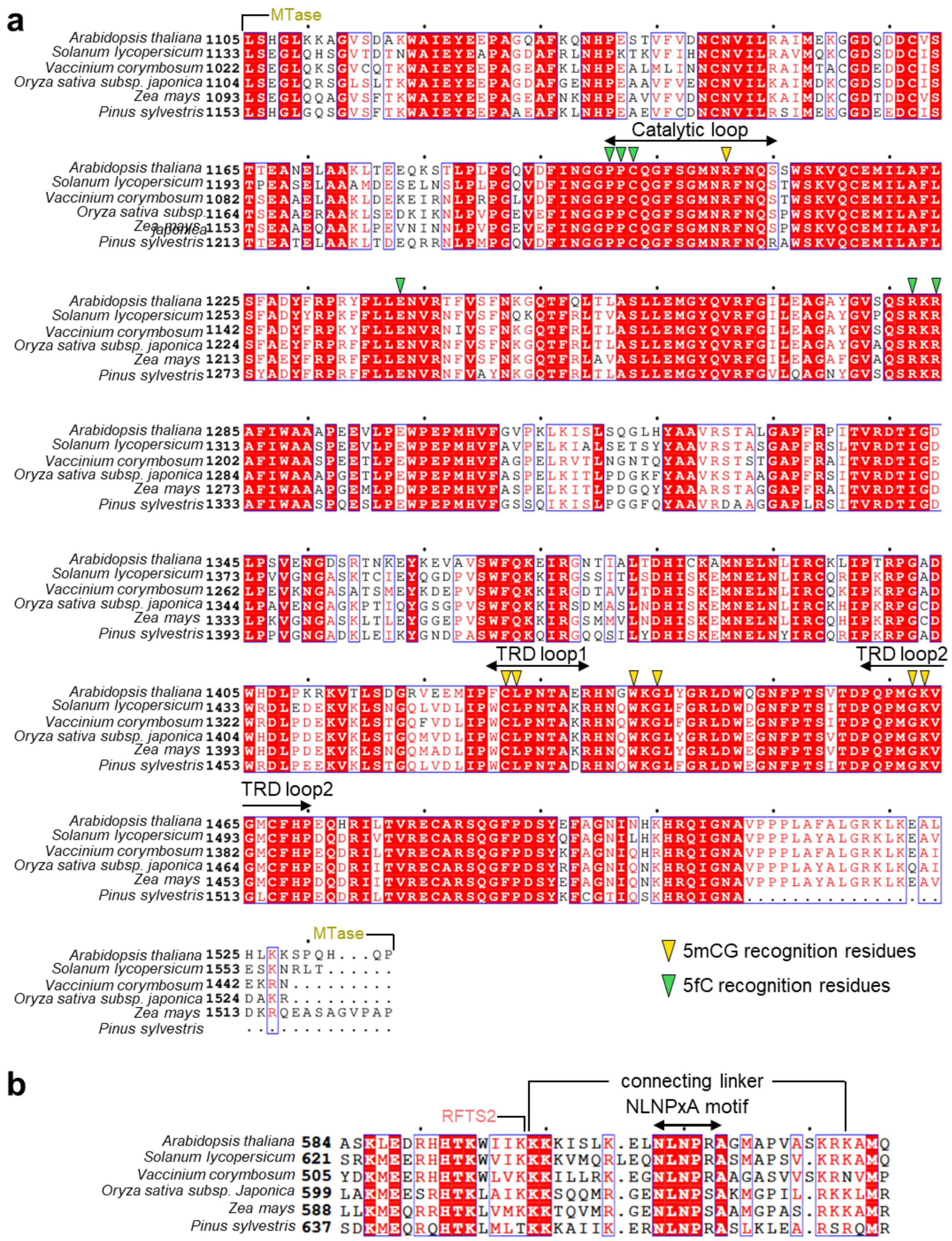

Supplementary Figure 4: Sequence alignment of MTase domains MET1 in plants.

Multiple sequence alignment of MET1 **(a)** MTase domain and **(b)** connecting linker in plants. The amino acid residues for recognition of 5mCG and 5fC are highlighted yellow and green arrows, respectively.

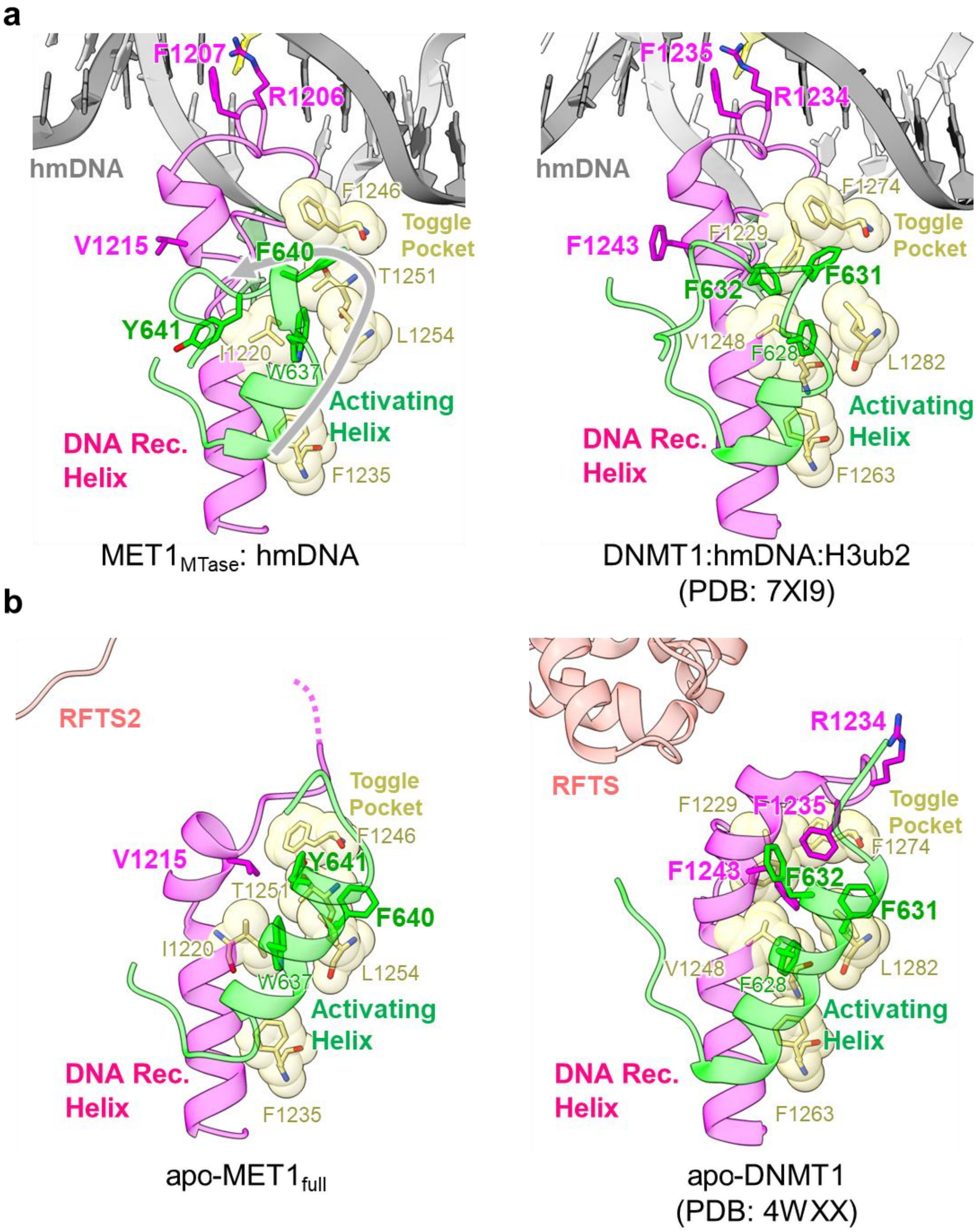

Supplementary Figure 5: Structural comparison of Activating Helix and Recognition Helix.
(a) Structures of the Toggle Pocket, shown as yellow sticks with transparent spheres, of MET1 (left) and DNMT1 (right, PDB: 7XI9) bound to hmDNA, in which the DNA Recognition Helix and Activating Helix are shown as magenta and green cartoons, respectively. hmDNA is shown as a gray cartoon. **(b)** Structures of the Toggle Pockets of apo-MET1$_{full}$ (left) and apo-DNMT1 (right, PDB: 4WXX). The RFTS2 domain in MET1 and RFTS domain of DNMT1 are shown as salmon cartons.

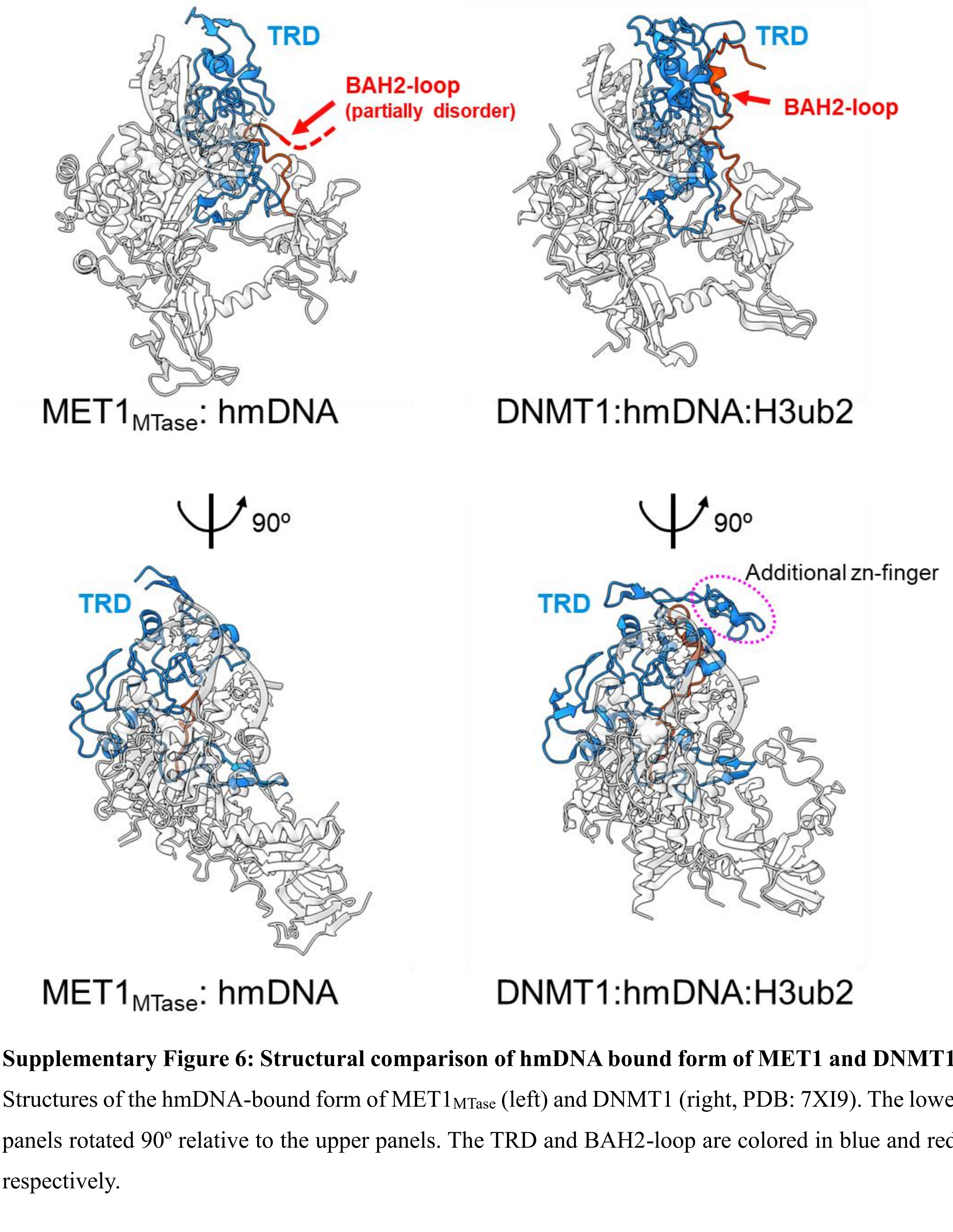

Supplementary Figure 6: Structural comparison of hmDNA bound form of MET1 and DNMT1. Structures of the hmDNA-bound form of MET1$_{MTase}$ (left) and DNMT1 (right, PDB: 7XI9). The lower panels rotated 90º relative to the upper panels. The TRD and BAH2-loop are colored in blue and red, respectively.

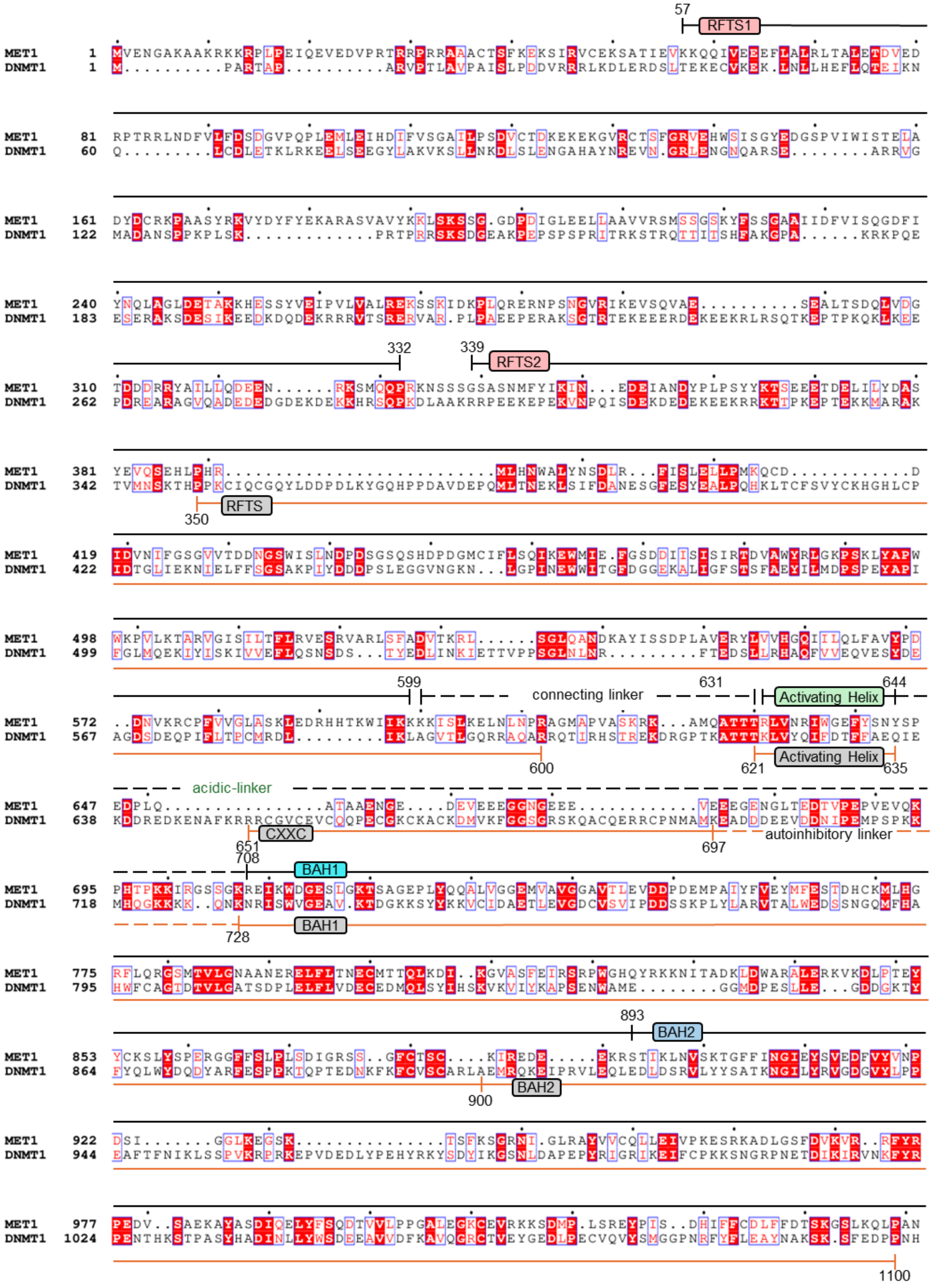

57
RFTS1
MET1
DNMT1
332
339
RFTS2
RFTS
350
599
connecting linker
631
Activating Helix
644
600
621
635
acidic-linker
CXXC
651
697
autoinhibitory linker
708
BAH1
728
893
BAH2
900
1100

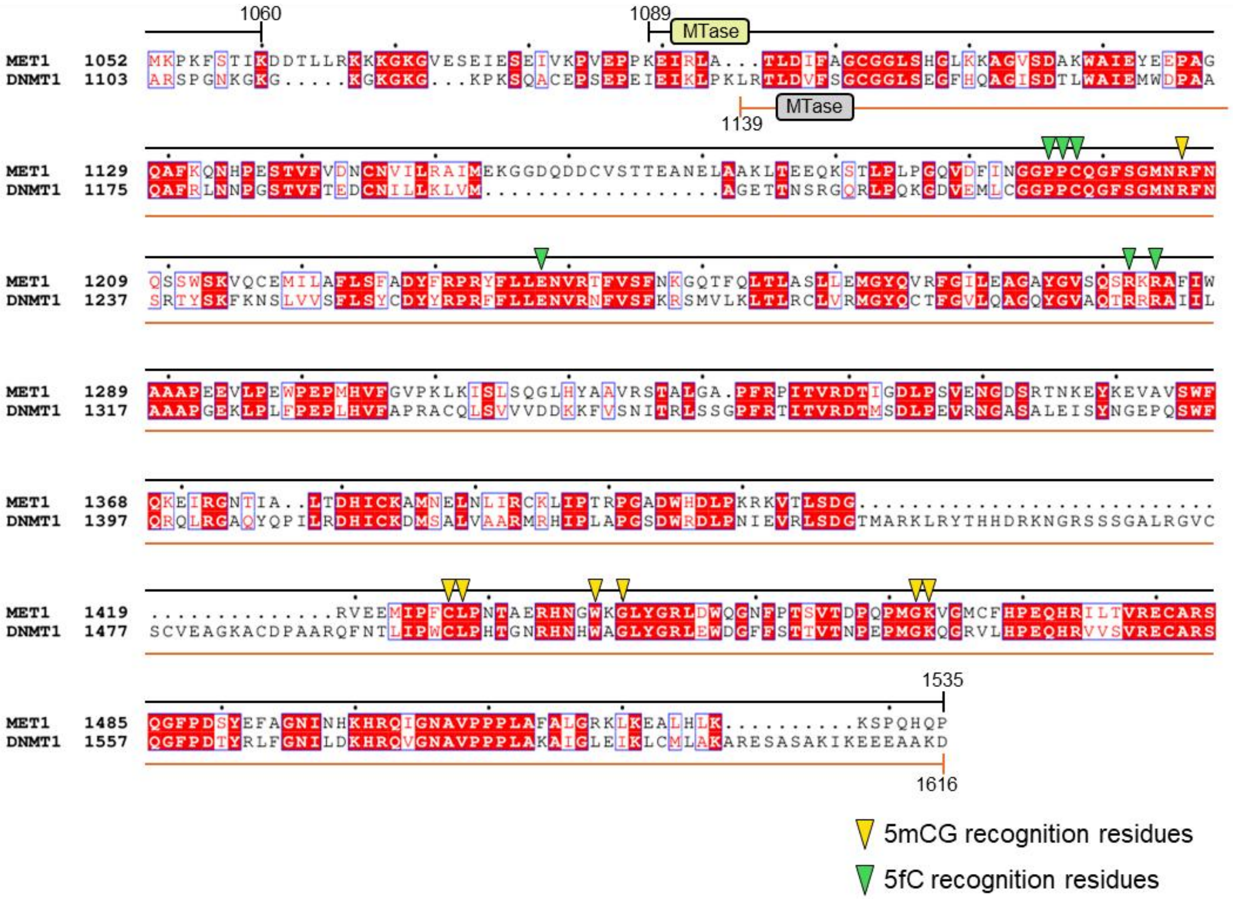

Supplementary Figure 7: Sequence alignment of *Arabidopsis thaliana* MET1 and human DNMT1.

Identical or similar amino acid residues are enclosed in boxes, and conserved residues are highlighted in red. MET1 domains are labeled with a black line above the corresponding sequences, and DNMT1 domains are labeled with an orange line below the corresponding sequences. The amino acid residues for recognition of 5mCG and 5fC are highlighted yellow and green arrows, respectively.

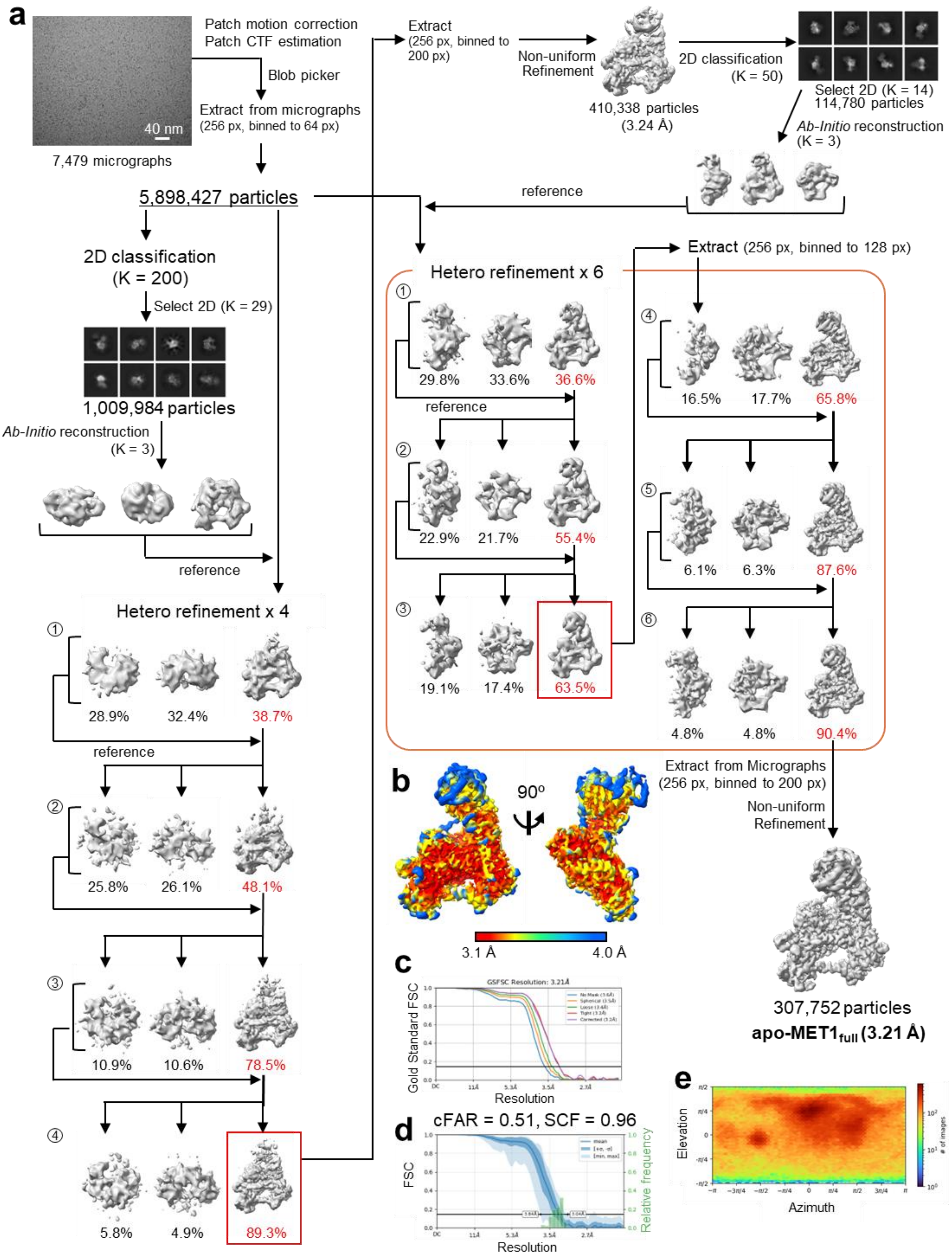
a
Patch motion correction
Patch CTF estimation
Blob picker
Extract from micrographs
(256 px, binned to 64 px)
40 nm
7,479 micrographs
5,898,427 particles
2D classification
(K = 200)
Select 2D (K = 29)
1,009,984 particles
Ab-Initio reconstruction
(K = 3)
reference
Hetero refinement x 4
①
28.9%
32.4%
38.7%
reference
②
25.8%
26.1%
48.1%
③
10.9%
10.6%
78.5%
④
5.8%
4.9%
89.3%
Extract
(256 px, binned to 200 px)
Non-uniform
Refinement
410,338 particles
(3.24 Å)
2D classification
(K = 50)
Select 2D (K = 14)
114,780 particles
Ab-Initio reconstruction
(K = 3)
reference
Hetero refinement x 6
①
29.8%
33.6%
36.6%
reference
②
22.9%
21.7%
55.4%
③
19.1%
17.4%
63.5%
Extract (256 px, binned to 128 px)
④
16.5%
17.7%
65.8%
⑤
6.1%
6.3%
87.6%
⑥
4.8%
4.8%
90.4%
Extract from Micrographs
(256 px, binned to 200 px)
Non-uniform
Refinement
307,752 particles
apo-MET1full (3.21 Å)
b
90°
3.1 Å
4.0 Å
c
GSFSC Resolution: 3.21Å
Gold Standard FSC
Resolution
d
cFAR = 0.51, SCF = 0.96
FSC
Relative frequency
Resolution
e
Elevation
Azimuth

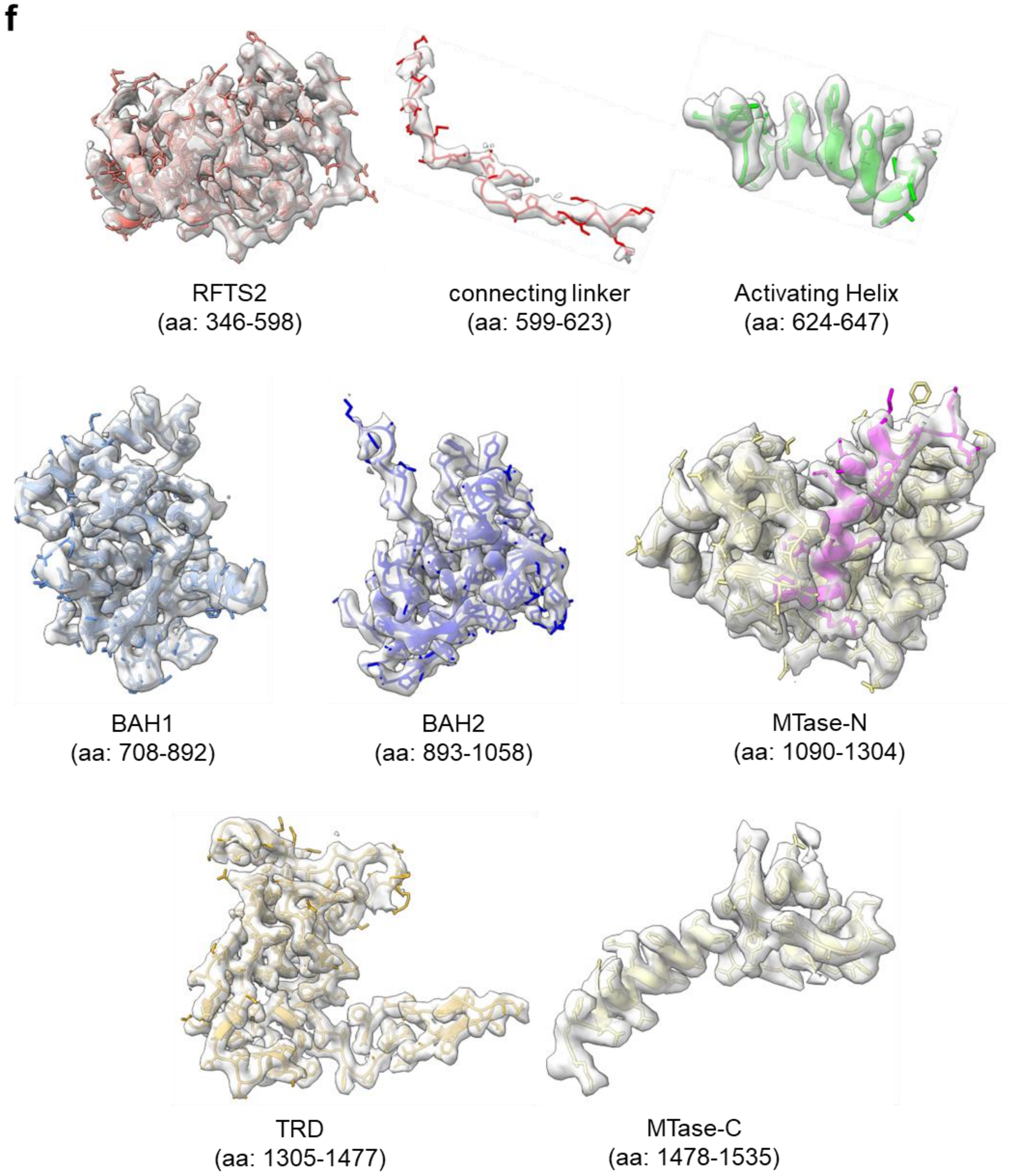

Supplementary Figure 8: Overview of cryo-EM data processing workflow for apo-MET1$_{\mathbf{full}}$.

(a) Data processing workflow performed using cryoSPARC. **(b)** Local resolution map. Resolution ranges are shown in box. **(c)** FSC curve. Resolution is reported at the FSC threshold of 0.143. **(d)** Directional resolution and Fourier sampling of the cryo-EM map. **(e)** The orientation distribution of the refined particles. **(f)** Cryo-EM maps of apo-MET1$_{full}$. The atomic models of the MET1 domains are superimposed on the semi-transparent cryo-EM map (gray).

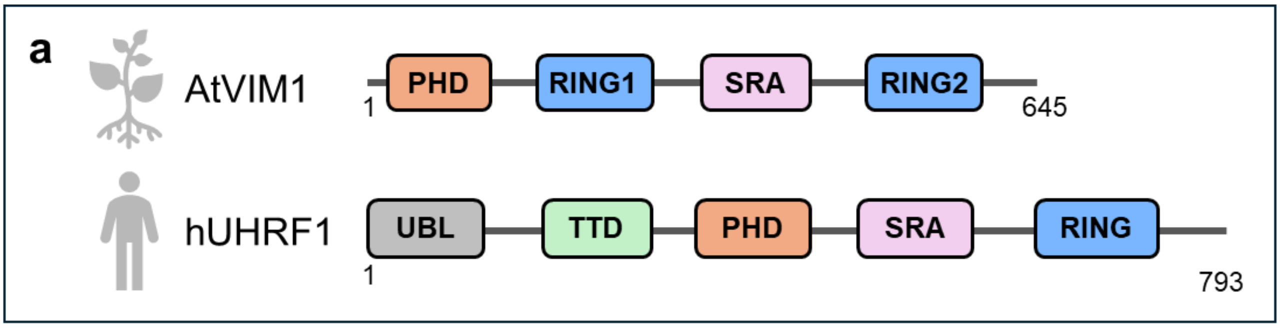

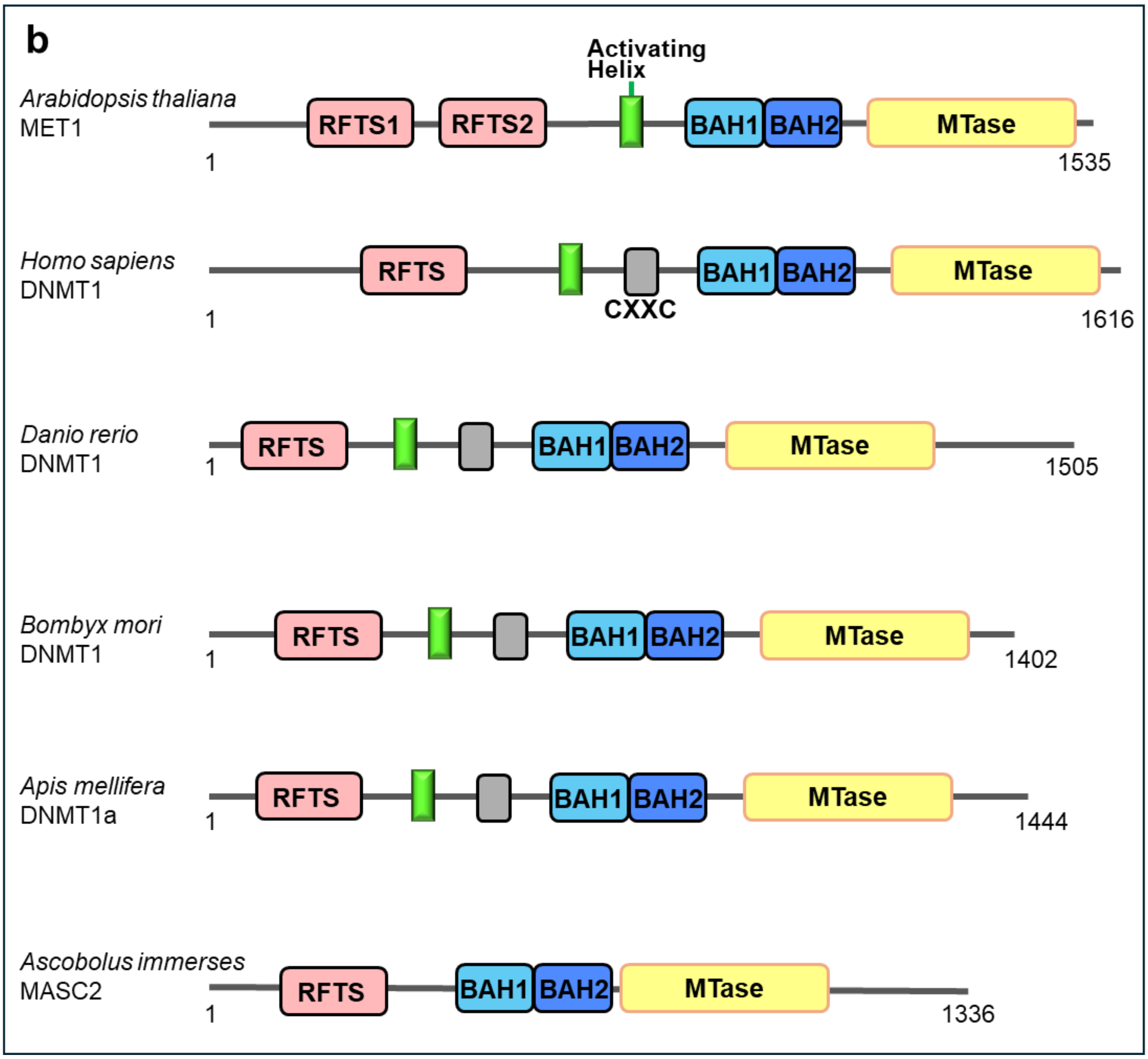

Supplementary Figure 9: Domain structures of VIM1, UHRF1 and various species of CG maintenance methyltransferase.

(a) Domain compositions of *Arabidopsis thaliana* VIM1 (AtVIM1, top) and human UHRF1 (hUHRF1, bottom). **(b)** Comparison of domain composition of *Arabidopsis thaliana*, *Homo sapiens*, *Danio rerio*, *Bombyx mori*, *Apis mellifera*, and *Ascobolus immerses* CG maintenance methyltransferases.

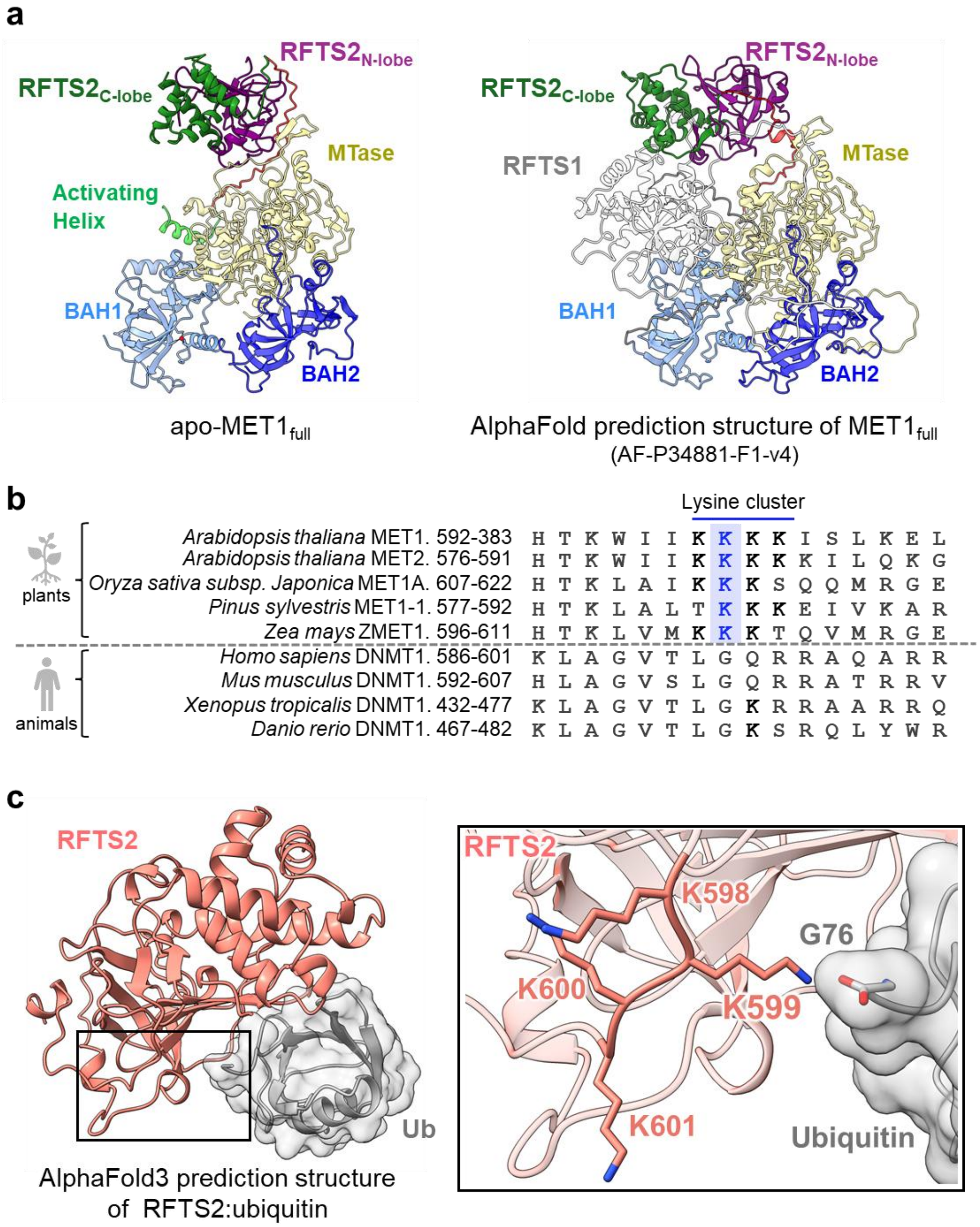

Supplementary Figure 10: Structural prediction of MET1.

(a) Comparison of the apo-MET1$_{full}$ (left) and AlphaFold-predicted structure of MET1$_{full}$ deposited in the AlphaFold Protein Structure Database (right).

(b) Multiple sequence alignment of CG DNA methyltransferases around the lysine cluster of MET1 in plants and animals. Lys599 of MET1 and the corresponding residues in other plants are highlighted.

(c) AlphaFold3-predicted structure of the RFTS2:ubiquitin complex. The RFTS2 domain of MET1 and ubiquitin are shown as salmon cartoon and gray cartoon with transparent surface, respectively. The right panel presents the putative isopeptide bond between Lys599 of MET1 and Gly76 of ubiquitin.

Supplementary Table 1:

Cryo-EM data collection, refinement and validation statistics

	$MET1_{MTase}$:hmDNA (EMDB-63650) (PDB 9M5U)		Apo-$MET1_{full}$ (EMDB-63652) (PDB 9M5X)
**Data collection and processing**	CHAPSO-	CHAPSO+	
Magnification	105,000		105,000
Voltage (kV)	300		300
Electron exposure (e^-/Å^2)	55.2	60.7	54.1
Defocus range (μm)	-0.8 to -1.6		-0.8 to -1.6
Pixel size (Å)	0.83		0.83
Symmetry imposed	*C*1		*C*1
Initial particle images (no.)	5,516,893	10,333,570	5,898,427
Final particle images (no.)	864,086		307,752
Map resolution (Å)	2.74		3.17
FSC threshold	0.143		0.143
Box size (px)	230		200
Voxel size (Å/px)	0.92		1.06
SCF[a]	0.91		0.96
cFAR[b]	0.66		0.51
**Refinement**			
Initial model used (AlphaFoldDB)	AF-P34881-F1		$MET1_{MTase}$:hmDNA
Model resolution (Å)	2.84		3.36
FSC threshold	0.5		0.5
Map sharpening *B* factor (Å^2)	-100		-100
Model composition			
Non-hydrogen atoms	6720		8259
Protein residues	783		1036
Nucleotide	24		-
Ion (zinc)	1		1
SAH	1		-
B factors (Å^2)			
Protein (min/max/mean)	9.34/77.77/31.24		47.53/259.09/155.30
Nucleotide (min/max/mean)	24.24/28.95/26.54		-
Ligand (min/max/mean)	22.14/82.87/50.07		204.77/204.77/204.77
CC (volume/mask)	0.84/0.87		0.76/0.75
R.m.s. deviations			
Bond lengths (Å)	0.002		0.002
Bond angles (°)	0.482		0.539
Validation			
MolProbity score	1.60		1.80
CaBLAM outliers	1.34		2.62
Clashscore	3.96		8.14
Poor rotamers (%)	3.29		0.00
Rama-Z score	0.19		0.08
EMRinger score	3.27		2.33
Ramachandran plot			
Favored (%)	97.91		94.87
Allowed (%)	2.09		5.13
Disallowed (%)	0.00		0.00

[a]: sampling compensation factor

[b]: conical FSC (Fourier shell correlation) area ratio

Supplementary Table 2:

Primer sequences used in this study

Primer name	Sequence
BacMET1-1F	GGGCCCCCAACGACCATGGTGGAAAATGGGGCTAAAGCTGCGAA
BacMET1-1535R	TATGGCTGATTACTACTAGGGTTGGTGTTGAGGAGACTTCTTGA
MET1-57F	GGGCCCCTGGGATCCAAGAAACAGCAGATTGTGGAGGAAGAGTT
MET1-332R	TCACGATGCGGCCGCCTAGGGCTGTTGCATAGATTTCCTATTCT
MET1_RFTS2-339F	GGGCCCCTGGGATCCGGTTCTGCTTCAAATATGTTCTACATTAA
MET1_RFTS2-616R	TCACGATGCGGCCGCCTATGCCATGCCTGCCCTTGGATTCAGAT
MET1-621F	GGGCCCCTGGGATCCAAGAGGAAAGCTATGCAAGCAACAACAAC
MET1-F640AY641A-F	GGGAGAGGCTGCCTCCAATTACTCTCCAGA
MET1-F640AY641A-R	AATTGGAGGCAGCCTCTCCCCAAATTCTGT
MET1_W1438A-F	CAACGGTGCGAAGGGACTATATGGGAGATT
MET1_W1438A-R	GTCCCTTCGCACCGTTGTGGCGCTCAGCTG
MET1_W637A-F	CAGAATTGCGGGAGAGTTTTACTCCAATTA
MET1_W637A-R	ACTCTCCCGCAATTCTGTTGACCAGGCGAG
MET1_M1204A-F	TTCTGGTGCGAACAGGTTCAACCAAAGCTC
MET1_M1204A-R	ACCTGTTCGCACCAGAAAATCCCTGACATG
MET1_R1206A-F	TATGAACGCGTTCAACCAAAGCTCTTGGAG
MET1_R1206A-R	GGTTGAACGCGTTCATACCAGAAAATCCCT
MET1_F1207A-F	GAACAGGGCCAACCAAAGCTCTTGGAGTAA
MET1_F1207A-R	TTTGGTTGGCCCTGTTCATACCAGAAAATC
6PVIM1-1F	GGGCCCCTGGGATCCATGGCGCGTGACATCCAACTCCCCTGCGA
6PVIM1-645R	TCACGATGCGGCCGCTCACCTGATGGTCGCAGAAACTGTTGCGT
UBC11-1F	GGGCCCCTGGGATCCATGGCTTCTAAGAGGATCTTGAAGGAGCT
UBC11-149stop-R	TCACGATGCGGCCGCTCAACCCATTGCGTACTTTTGTGTCCAGC

* All primers used in this study were custom-designed and synthesized by Eurofins Genomics, Japan.
